# Clarifying the Concepts of Personalization and Tailoring of eHealth Technologies: Multimethod Qualitative Study

**DOI:** 10.2196/50497

**Published:** 2024-11-13

**Authors:** Iris ten Klooster, Hanneke Kip, Sina L Beyer, Lisette J E W C van Gemert-Pijnen, Saskia M Kelders

**Affiliations:** 1 Centre for eHealth and Wellbeing Research Department of Psychology, Health & Technology University of Twente Enschede Netherlands; 2 Department of Research Stichting Transfore Deventer Netherlands; 3 Optentia Research Unit North-West University Vanderbijlpark South Africa

**Keywords:** eHealth, personalization, tailoring, segmentation, adaptation, interviews, definition

## Abstract

**Background:**

Although personalization and tailoring have been identified as alternatives to a “one-size-fits-all” approach for eHealth technologies, there is no common understanding of these two concepts and how they should be applied.

**Objective:**

This study aims to describe (1) how tailoring and personalization are defined in the literature and by eHealth experts, and what the differences and similarities are; (2) what type of variables can be used to segment eHealth users into more homogeneous groups or at the individual level; (3) what elements of eHealth technologies are adapted to these segments; and (4) how the segments are matched with eHealth adaptations.

**Methods:**

We used a multimethod qualitative study design. To gain insights into the definitions of personalization and tailoring, definitions were collected from the literature and through interviews with eHealth experts. In addition, the interviews included questions about how users can be segmented and how eHealth can be adapted accordingly, and responses to 3 vignettes of examples of eHealth technologies, varying in personalization and tailoring strategies to elicit responses about views from stakeholders on how the two components were applied and matched in different contexts.

**Results:**

A total of 28 unique definitions of tailoring and 16 unique definitions of personalization were collected from the literature and interviews. The definitions of tailoring and personalization varied in their components, namely adaptation, individuals, user groups, preferences, symptoms, characteristics, context, behavior, content, identification, feedback, channel, design, computerization, and outcomes. During the interviews, participants mentioned 9 types of variables that can be used to segment eHealth users, namely demographics, preferences, health variables, psychological variables, behavioral variables, individual determinants, environmental information, intervention interaction, and technology variables. In total, 5 elements were mentioned that can be adapted to those segments, namely channeling, content, graphical, functionalities, and behavior change strategy. Participants mentioned substantiation methods and variable levels as two components for matching the segmentations with adaptations.

**Conclusions:**

Tailoring and personalization are multidimensional concepts, and variability and technology affordances seem to determine whether and how personalization and tailoring should be applied to eHealth technologies. On the basis of our findings, tailoring and personalization can be differentiated by the way that segmentations and adaptations are matched. Tailoring matches segmentations and adaptations based on general group characteristics using if-then algorithms, whereas personalization involves the direct insertion of user information (such as name) or adaptations based on individual-level inferences. We argue that future research should focus on how inferences can be made at the individual level to further develop the field of personalized eHealth.

## Introduction

### Background

eHealth technologies can provide opportunities to overcome the increased burden on health care [1]. For example, they can provide a more cost-efficient approach and improve the quality of care by exploiting the additional capabilities of technology such as continuous monitoring and allowing patients to move in a virtual world [2]. eHealth can be defined as “the use of technology to improve health, well-being, and health care” [2], and it is an umbrella term that encompasses a variety of digital health care technologies, such as mobile health apps, web-based monitoring systems, and web-based interventions [3]. Although eHealth technologies show significant improvements in health and well-being, these improvements are often small [4-7], adherence and engagement are regularly low [8], and the effectiveness seems to decline in the long term. This suggests that the full potential of eHealth technologies has not been realized. Because eHealth technologies that adapt to individuals are associated with more effective interventions [9-11], a possible explanation for the suboptimal effectiveness is that a “one-size-fits-all” approach is not sufficient. This means that the design of an eHealth technology needs to consider the variation in patients’ needs, life experiences, and other factors [12]. For example, eHealth technologies focused on stress management appeared to be effective for those reporting increased stress, whereas this type of intervention is not effective when used by all workers [5]. While the adaptations of health care are largely based on information about the medical characteristics of the patient (eg, genes) [13], advances in eHealth technologies are creating new opportunities to continuously collect more holistic data about a patient. For example, information on stress levels might be obtained using ecological momentary assessment or wearables connected with the eHealth technology. Changing the eHealth technology according to the individual user is thought to increase effectiveness and implementation. This is referred to as tailoring and personalization in the context of eHealth technologies. Personalization and tailoring seem to be logical ways to overcome suboptimal effectiveness, but there is no clear agreement on how to define personalization and tailoring and what the differences and similarities are. In addition, it remains unclear how personalization and tailoring are being applied and can be applied to eHealth technologies. We need to understand more about personalization and tailoring to maximize their impact and move a step closer to realizing the full potential of eHealth technologies.

### Personalization and Tailoring

What tailoring and personalization have in common is that certain adaptations are made for the individual user [14-16], although definitions vary in their operationalization. In the field of health communication, the focus is on presenting information that is perceived as relevant to the user, thereby increasing their engagement and, in turn, leading to improved behavior change outcomes according to the Elaboration Likelihood Model [17]. An example of personalization in this area is the inclusion of the user’s name (identification) [18], while a broader definition includes other strategies, such as contextualization (eg, “This information is relevant to mothers.”) and raising expectations (eg. “This information is especially designed for you.”) [15]. These strategies merely focus on communicating that the information is relevant to the user, whereas the persuasive system design principles operationalize personalization as the actual provision of relevant information [16]. This does not necessarily involve explicitly stating that the information is relevant to the user, but the actual delivery of relevant information may indirectly be perceived as more relevant and reduce the burden on the user by excluding irrelevant information. An example of this operationalization is providing arguments for why eating fruit is important only to the users who have indicated that they do not eat fruits. A more recent example is adapting the mode of delivery of health communication [19].

In addition to health communication, personalization and tailoring are also used in other areas of research. An example is a study on customization and personalization of a dating website [20], where tailoring is operationalized as providing dating recommendations based on one’s own qualities (personalization) or explicit preferences (customization). Another example is a study that investigates the effectiveness of web-based advertising when the advertisements are adapted to the preferences compared to location-based adaptations (showing advertisements for nearby businesses). Another example is in e-commerce, where cluster analysis is used to build user profiles that are used for real-time personalization [21].

Tailoring is sometimes described as an umbrella term for personalization, feedback, and content matching [15]. According to this definition, personalization is a form of tailoring. However, tailoring is also defined as a term that is distinct from personalization. Oinas-Kukkonen and Harjumma [16] distinguish personalization and tailoring by considering adaptations to groups of users as tailoring and to individual users as personalization. This contrasts with the definition by Revere and Dunbar [18], which sees tailoring as adaptations to individual users and adaptations to groups of users as a form of targeting eHealth technologies. Overall, we find that several attempts have been made to describe personalization and tailoring unambiguously, but these definitions are sometimes contradictory and the understanding of the differences and similarities between these 2 concepts vary.

Several distinctions are made within the concepts of tailoring and personalization, such as first, second, and third generation; dynamic versus static; deep versus surface; self- versus computer-based; and manual versus algorithm-based. First, distinctions are made that describe how tailoring has evolved over time, referred to as first generation (print), second generation (multimedia), and third generation (mobile and handheld devices) [22]. Static and dynamic tailoring refers to whether data are collected once for segmentation and adapted accordingly (static) or whether data are collected multiple times over time (dynamic) [10]. The data may be behavioral data or observable factors (surface tailoring), determinants of health behavior (deep tailoring), or a combination of these [23]. A further distinction is whether the user determines how the technology is customized or whether it is determined for the user. The terms personalized, automated, and authoritative refer to technologies that are adapted *for* the user, whereas customization, autonomous, and self-tailoring refer to technologies that are adapted *by* the user [24,25].

### Segmentation, Customization, and Matching

As mentioned earlier, it remains unclear how personalization and tailoring are applied to eHealth technologies. To this end, Hawkins et al [15] developed a framework in which the application of personalization and tailoring is described in terms of segmentation and customization. Segmentation is “the degree to which the audience is divided into increasingly more defined, homogenous groups,” a concept that originated in marketing [26]. Segmentation takes place based on user characteristics such as lifestyle and the ability to understand health information or health status, creating groups ranging from very small (one person) to large (all women). Furthermore, based on these segmentations, customization of the content or design of an eHealth technology takes place [15]. Customization is described as “the degree to which the messages (ie, a combination of content, source, graphics, channel, etc) that the audience receives reflect relevant individual characteristics” [15]. In this study, we refer to what Hawkins called “customization” as “adaptation” to avoid confusion about how customization is defined in the field of design research and IT [24,27]. A meaningful link between segmentation and adaptation results in a personalized or tailored eHealth technology, which can be described on a continuum of segmentation and adaptation. A limitation of this model is that it only focuses on messages, whereas advances in eHealth technologies expand how they can be adapted. This means that the focus of message adaptation can, and should, be extended to, for example, changing virtual environments and intensity levels of exercise in a gamified sports game. Another limitation of this model is that it does not describe how segmentation and adaptation can be linked in a meaningful way. In this study, we refer to this aspect of creating a meaningful link as “matching.”

### Aims of This Study

The goals of this study are to describe in detail (1) how tailoring and personalization are defined according to eHealth experts and in literature and what the differences and similarities are, (2) on the basis of what type of variables users are segmented to allow for adapting of eHealth technologies, (3) what elements of eHealth technologies are adapted to those segments, and (4) how the segments are matched with eHealth adaptations.

## Methods

### Study Design

This study used a multimethod qualitative study design to explore the definitions, segmentation, and adaptation strategies used in personalized and tailored eHealth technologies. The study used semistructured interviews incorporating vignettes with individuals who work in eHealth, alongside an analysis of definitions of personalization and tailoring from references cited in eHealth evaluation studies.

### Interviews

#### Participants

Participants were researchers who were fluent in English, who work or have been working with eHealth technologies, and who published at least 1 scientific article in the field of personalized or tailored eHealth technologies. They were included via purposive and snowball sampling by asking participants to identify researchers with expertise in personalizing and tailoring eHealth technologies. The participants were approached via email and received an information sheet about the interviewer’s background and the purpose of the study, including the framework by Hawkins et al [15] to provide context. In total, 10 participants consented to participate in the interviews, of whom there were 4 (40%) men, 2 (20%) women, and 1 (10%) nonbinary participant (n=3, 30% participants did not disclose their gender). Their mean age was 39 years (SD 5.93 y; 4 missing values), and the majority (n=4, 40%) worked as a professor. Other professions were associate professor, professor emeritus, lecturer, and physiologist.

#### Materials and Procedure

The interviews were developed based on the framework proposed by Hawkins et al [15] and piloted among 2 individuals to ensure clarity of the questions. Feedback from the pilot interviews was used to refine the interview protocol. The interviews took place on the web to adapt to the COVID-19 restrictions, had a duration of around 60 minutes, and were video recorded after receiving permission from the participants. After a short introduction, questions were asked in two parts, namely (1) general questions about personalization and tailoring and (2) questions related to vignettes with 3 examples of eHealth technologies, varying in personalization and tailoring strategies (Multimedia Appendix 1 provides the full interview scheme). General questions were related to definitions of personalization and tailoring (eg, “Do you think there are differences between personalization and tailoring or any other similar terms, such as targeted, individualized, adapted?”), questions about how participants segmented users in their projects (eg, “How would you segment the users of eHealth technologies to provide them with personalized content?”), their experience with adapting eHealth technologies (eg, “How can eHealth technologies be customized to the end user?”), and how these 2 were matched (eg, “How do you think customization and segmentation are related?”).

The second part of the interviews consisted of 3 vignettes to stimulate a reaction or opinion of the participant toward the depicted situation on the vignette. The vignette depicted 3 eHealth technologies that differ in their level of segmentation and the way in which the technologies are adapted. The first vignette is from the Brain Aging Monitor study [28], in which users are segmented into lifestyle profiles. Adaptations to those lifestyle profiles include feedback about lifestyle and the inclusion or exclusion of relevant content. The second vignette consists of a virtual reality (VR) behavior therapy for tobacco cessation [29], in which users are segmented into personas based on emotional response data. Adaptations entail the use of either positive (ie, cessation coach) or negative scenarios (ie, receiving a diagnosis of emphysema). The third vignette is a cardiovascular risk calculator [30], in which users are segmented based on several cardiovascular risk factors. The eHealth technology is adapted by indicating their cardiovascular risk and whether they are normal weight, overweight, or obese. The participants were introduced to a vignette and were asked to identify the aspects of the eHealth technology that are personalized to the user as well as how they would further segment and adapt the eHealth technology themselves.

The interviewer (SLB) was a female BSc student who received education in conducting qualitative research. There was no prior relationship established with the participants apart from the recruitment and planning required. The interviews took place with only the interviewer and the interviewee present. The COREQ (Consolidated Criteria for Reporting Qualitative Research) checklist [31] can be found in Multimedia Appendix 2.

### Ethical Considerations

Ethics approval for this study was obtained from the ethics committee of the Faculty of Behavioral, Management, and Social Sciences of the University of Twente (request number 210375). All participants signed informed consent before the interviews, and they could opt out at any time. In such cases, no further data would be collected, and any data already collected would be deleted. Before analysis, the data were anonymized, and identifiable information was removed from the transcriptions (eg, when the participant mentioned names). The participants did not receive any compensation for participating in the study.

### Literature

#### Search Strategy

For this study, we used definitions extracted from our earlier comprehensive review of personalization approaches in eHealth evaluation studies. The full review is reported elsewhere [32]. In the systematic review, we conducted an electronic search through the databases Scopus, PubMed, Embase, PsycINFO, and IEEE Xplore. We included peer-reviewed journal articles and conference papers describing personalized eHealth evaluation studies in which participants were randomly assigned to their condition, and outcomes were related to health or well-being. In addition, we only included studies that described how segmentations were matched with adaptations in a computerized way and with full text in German, English, or Dutch. In this study, we specifically used the definitions of personalization and tailoring cited in studies included in our review, along with the original references to these definitions as provided by those included studies. These definitions served as a foundation to explore expert perspectives through qualitative interviews, ensuring a distinct focus that builds on but does not substantially overlap with the previous work.

#### Data Extraction

Data were extracted from the definitions of tailoring and personalization used in the eHealth evaluation studies that were included. References to their definitions were collected. As a subsequent step, we extracted the definitions from the literature they referred to. By extracting these references, we aimed to identify key elements of definitions that are used in eHealth. References that did not provide a definition were excluded, and as a final step, only the unique definitions were selected.

### Data Analysis

The interview data were transcribed manually and anonymized for analysis using Microsoft Excel. Reflexive thematic analysis, as described by Braun and Clarke [33] and further elaborated in their 2023 publication [34], was used to analyze the interview data, incorporating the reactions to the vignettes and the published definitions. This approach emphasizes the active role of the researcher and the interpretative nature of thematic analysis. Our analysis combined deductive and inductive coding strategies. Initially, deductive coding was used to identify fragments related to the research questions—definition, segmentation, adaptation, and matching—based on existing literature and theoretical frameworks. Subsequently, inductive coding allowed the identification of themes that emerged directly from the data without being constrained by predefined categories. Following this, these initial codes were merged into themes, and definitions and names for the themes were generated [35]. This dual approach ensured a comprehensive analysis that incorporated both preexisting knowledge and new insights from the data. To ensure reliability, 1 interview was coded by 2 researchers (HK and ITK), and the interrater reliability was 0.93 for the themes. This step was not only about measuring interrater reliability but also about engaging in a reflexive dialogue. Differences in coding perspectives were discussed, and changes to the thematic scheme were made accordingly, leading to a deeper understanding of the data. The resulting themes were reported with quotes from the interviews for illustration so the reader can confirm our interpretations.

## Results

### Definitions

Of the 412 eHealth evaluation studies included, 104 (25.2%) studies defined tailoring or personalization by referencing definitions from previous research. Of the 71 (68.3%) studies that referred to tailoring definitions, there were 20 (28.2%) unique references. In addition, of the 33 (31.7%) studies that referred to personalization definitions, there were 6 (18.2%) unique references.

eHealth evaluation studies referring to tailoring definitions mainly referenced to de Vries and Brug [36] (n=15, 21%), Kreuter and Wray [37] (n=11, 16%), Kreuter et al [38] (n=8, 11%), and Hawkins et al [15] (n=8, 11%). The unique definitions of tailoring with the number of references to these definitions in eHealth evaluation studies can be found in Multimedia Appendix 3 [10,15,36-52]. In Table 1 the themes that we identified in the published definitions and definitions according to eHealth experts are summarized to identify key elements.

A total of 10 (36%) of the 28 definitions include the term *adaptation*, referring to adapting health education materials [36]; the provision of support [39]; adjusting message frames [40] and information [41]; customizing the source, message, and channel of a given communication [37]; and mimicking to some extent a classic tailoring technique, that of “person-to-person” counseling [42-44].

In 16 definitions, the term *individual* appeared, ranging from adaptations for the individual [15,36-38,43] to collecting data from individual users [10,39,42,44-48]. *User groups* appeared in 5 (17.9%) definitions of tailoring, such as “needs, interests, personality, usage context, or other factors relevant to a user group” [16], and 1 definition included the way that the tailoring strategy is developed:

...tailored medicine, then you’re still looking for those outcomes of randomized control trials and try to adjust.

Participant 6

Several definitions included the segmentation variables for tailoring, namely *preferences* (7/28, 25% definitions), *symptoms* (2/28, 7% definitions), *characteristics* (9/28, 32% definitions), *context* (4/28, 14% definitions), and *behavior* (2/28, 7% definitions). In total, 20 (71%) of the 28 definitions included customizations (content, identification, feedback, channel, or design). Content and channeling were mainly included in the definitions of tailoring. Definitions that included content-related customizations were related to changes in health education materials [36], communications [10,15,37], information [16,38,42], health messages [49], health information [50], advice [43], individual treatment plans, behavior change plans, or dietary advice [44], information on health behavior change [51], content [45,52], treatment [46], messages [47,48], persuasive text [48], and to “what kind of messages you give what kind of information” (participant 2). Definitions that included channeling were related to adapting source and channel [37]; amount, type, and through channels of delivery [50]; the way that the content is presented [45]; the presentation modality [41]; or highlighting some bits of information while omitting others [40]. One definition did not concern adapting the channel but mentioned that tailored content can be presented through different channels [48].

In total, 8 (29%) of the 28 definitions included terms related to computerization, such as “computerized” [36,42], “decision rules” [10,49] or “automatically” [10], “expertise programmed into a computer” [44], and “algorithm” [47]. Outcomes were included in 8 (29%) definitions of tailoring, and these ranged from increasing relevance [15,37,42], persuasiveness [16,49], enhancing motivation [50], attention [50], reducing barriers to exposure [50], widely distributing at low cost [43], and reaching the target group (participant 10).

The unique definitions of personalization can be found in Multimedia Appendix 4 [15,36,53-55]. Table 2 summarizes the themes that we identified in the definitions of personalization in the literature and according to eHealth experts to identify key elements.

**Table 1:** Themes identified in definitions of tailoring.

Study and participant		Adaptation	Individuals	User groups	Preferences	Symptoms	Characteristics	Context	Behavior	Content	Identification	Feedback	Channel	Design	Computerized	Outcomes
**Study**																
	de Vries and Brug [36], 1999	✓	✓							✓					✓	
	Kreuter and Wray [37], 2003	✓	✓							✓			✓			✓
	Kreuter et al [38], 2000		✓				✓			✓						
	Hawkins et al [15], 2008		✓							✓						✓
	Krebs et al [10], 2010		✓							✓		✓			✓	
	Lustria et al [49], 2009									✓					✓	✓
	Brug et al [42], 2003	✓	✓				✓			✓					✓	✓
	Oinas-Kukkonen and Harjumma [16], 2009			✓	✓		✓	✓		✓						✓
	Rimer and Kreuter [50], 2006					✓				✓			✓	✓		✓
	Kroeze et al [43], 2006	✓	✓				✓	✓	✓	✓						✓
	Brug et al [44], 1999	✓	✓							✓		✓			✓	
	Smeets et al [51], 2007		✓							✓						
	Bental et al [45], 1999		✓		✓					✓			✓			
	Nahum-Shani et al [39], 2016	✓	✓				✓	✓								
	Păsărelu et al [46], 2017		✓		✓	✓	✓			✓						
	Strecher [47], 1999		✓		✓		✓			✓					✓	
	Smit et al [40], 2015	✓			✓								✓			
	Nguyen et al [41], 2016	✓			✓								✓			
	Dijkstra [48], 2008		✓							✓	✓		✓			
	Heron and Smyth [52], 2010		✓					✓	✓	✓		✓	✓			
**Participant**																
	Participant 1				✓		✓									
	Participant 2			✓						✓						
	Participant 3														✓	
	Participant 5	✓	✓				✓				✓			✓		
	Participant 6	✓		✓												
	Participant 7			✓												
	Participant 8			✓												
	Participant 10															✓

**Table 2:** Themes in the definitions of personalization.

Study and participant		Adaptation	Individuals	User groups	Preferences	Symptoms	Characteristics	Context	Behavior	Content	Identification	Feedback	Channel	Design	Computerized	Outcomes
**Study**																
	de Vries and Brug [36], 1999										✓					
	Hawkins et al [15], 2008										✓					✓
	Dijkstra [53], 2005		✓								✓					
	Oinas-Kukkonen [16], 2009									✓						✓
	DiClemente et al [54], 2001		✓				✓					✓				
	Evers et al [55], 2014									✓						
**Participant**																
	Participant 1	✓														✓
	Participant 2	✓	✓													
	Participant 3							✓	✓	✓			✓			
	Participant 4							✓								
	Participant 5	✓	✓	✓						✓				✓		
	Participant 6	✓	✓													
	Participant 7		✓	✓						✓						
	Participant 8		✓								✓	✓				✓
	Participant 9			✓												
	Participant 10				✓											

Interestingly, the definitions from the literature did not include *adaptation*, whereas 4 participants included adaptations, such as “trying to come close to the efficacy of having only really a one-on-one chat” (participant 1), adapting a digital health intervention (participant 2), “As long as there is some adaptation going on that changes the product from generic to specific to an individual or group of individuals. It’s personalization to me.” (participant 5), “adjust or adapt your, maybe also your assessment and the treatment of patients in a way that it’s really fits that person” (participant 6).

The theme *individuals* appeared in 7 (44%) of the 16 definitions of personalization, such as “recognizable feature or set of features refers undeniably to the person, e.g. the person’s first name or the combination of objective behavioral features such as the number of cigarettes smoked, the number of years the person smoked, and the brand smoked” [53] or in another definition “personalized feedback represents the most individualized type of feedback” [54], and “helping people at an individual level to make healthy choices” (participant 8).

*User groups* did not appear in the definitions in the literature, while 3 (30%) of the 10 participants included this in their definitions. In 2 (12%) of the 16 definitions, participants mentioned that personalization entails both adaptations to individuals and user groups, while another definition described personalization as an adaptation to different stakeholders:

It is how to shape and design, intelligent design, technology to support the different stakeholders in health care.

Participant 9

Adaptations that were included in the definitions mainly referred to content and identification. An example of content is “offering specific physical, psychological, or social treatment modules instead of a standard treatment package” [55], “provide specific health information for women” (participant 5). Examples of identification included in the definitions are “listing a person’s name” [36], “conveying, explicitly or implicitly, that the communication is designed specifically for ‘you’” [15], “incorporating recognizable aspects of a person in a general text” [53], “the feedback that is being offered should be experienced as personal so that people think or experience or perceive that they are being spoken to in a personal way. It accounts to them and not per se to their fellow peers or something” (participant 8).

In total, 4 (25%) of the 16 definitions of personalization focused on the outcomes of personalization. These include “increase attention or motivation to process messages” [15], “greater capability for persuasion” [16], and “trying to come close to the efficacy of having only really a one-on-one chat, but at the same time making it available for a lot of people. So, the kind of this tradeoff is there” (participant 1), and “So, it’s a lot about the subjective experience, I would say. And it should lead to actionable insights that can be applied to their personal life” (participant 8).

### Segmentation

During the interviews, participants described 9 types of variables that can be used for segmenting eHealth users, namely demographic variables, preferences, health information, psychological variables, behavioral variables, individual determinants, environmental variables, intervention interaction, and technology variables (Table 3).

**Table 3:** Variable types for segmenting eHealth users (N=227).

Variables	Themes, n (%)	Participants, n (%)
Demographic	46 (20.3)	8 (80)
Preferences	43 (18.9)	8 (80)
Health	32 (14.1)	8 (80)
Psychological	25 (11)	8 (80)
Behavioral	25 (11)	5 (50)
Individual determinants	23 (10.1)	7 (70)
Environmental	20 (8.8)	4 (40)
Intervention interaction	9 (4.0)	4 (40)
Technology	6 (2.6)	4 (40)

Demographic variables were the most-mentioned variables (46/227, 20.3%). Examples of demographic variables that were mentioned are gender, age, education level, ethnicity, nationality, occupation, and place of residency. During the interviews, participants mentioned that demographic information largely consists of variables that are unchangeable, and therefore, other types of variables are preferably used for segmenting eHealth users.

Next to demographic variables, preferences were regularly mentioned as a variable for segmenting eHealth users (43/227, 18.9%). Behavioral preferences were related to whether eHealth users prefer different behaviors related to the target behavior of the eHealth system (eg, whether users like exercise or food and sport preferences). Participants also mentioned preferences that were related to the content of the eHealth technology. An example is to use the needs of the user for segmenting eHealth users:

People may themselves perceive themselves as, you know, maybe in need of some information, but not so much therapeutic strategies or changing lifestyle while from a professional or clinical perspective, that may be exactly what you want to offer to this specific person. Again, this is something that relates to perceived need.

Participant 10

Channeling preferences that were mentioned by the participants were related to preferences on how technology delivers the behavior change intervention. Examples are preferences on when to receive messages, how the users of eHealth technologies want to communicate with their health care provider, and preferences for communication styles. Next among the preferences, graphical preferences were mentioned as a variable that can be used for segmenting eHealth users (eg, preference for graphics within eHealth technology, color preferences, and layout and interface preferences).

Participants regularly mentioned health-related variables as a way to segment eHealth users (32/227, 14.1%). First, participants mentioned biomedical risk factors, such as BMI, cholesterol levels, blood pressure, and weight. Next to biomedical risk factors, participants mentioned medical conditions as a way to segment eHealth users (eg, type of diabetes). Medications taken by the eHealth user and the treatment process were also mentioned as variables for segmenting eHealth users. Psychological variables were mentioned by participants as a way to segment users of eHealth technologies (25/227, 11%). These variables were related to the eHealth user’s personality, well-being, psychological distress (eg, depression and anxiety), and emotions experienced by the user.

Behavioral variables that were mentioned by the participants (25/227, 11%) were related to things that can be observed from the outside, such as physical activity, sleeping, and extended sitting. Behavioral variables ranged from more specific behaviors (eg, steps and extended sitting) to more overarching behaviors that include >1 behavior (eg, lifestyle). Moreover, an example of a more indirect variable was weight behavior, which was used as an indication of whether the user was still engaged in losing weight.

Individual determinants mentioned by the participants were related to the users’ internal factors that determine behavior, such as attitudes, knowledge, and stage of change (23/227, 10.1%). Participants mainly mentioned determinants that were related to theories and models of behavior and behavior change. An example of a determinant was given by participant 1, who described that using determinants to segment eHealth users increases the potential to change someone’s behavior:

And if you personalize them on that then it almost doesn’t matter which gender you are, you know, it’s much closer to what you think about this topic. And you might be the female exception on this topic, or I may be the male exception. But if the content is tailored to what I think, I think that has much more potential there for change.

Participant 1

Participants also mentioned environmental variables (20/227, 8.8%) that are related to the surroundings of the eHealth user. Examples include time, place, exposure, and day of the week. More distant examples are culture and climate. An example of the place was given by participant 3:

Context is everything from the obvious, the obvious would be if you are outside of a McDonald’s restaurant, message on snack food might be beneficial. So that’s the low hanging fruit.

Participant 3

The user’s interaction with the eHealth technology was also mentioned as a way of segmenting eHealth users (9/227, 4%). This includes the more specific parts of how they interact with the system (eg, how often a particular feature is used) as well as the broader use characteristics (eg, adherence, adoption, and engagement). Technology-related factors were the least mentioned by participants (6/227, 2.6%) to segment users into smaller homogeneous groups. These variables include the extent to which the user is able or experienced in using different forms of technology. Examples of technology-related variables were related to how skilled or experienced users are with technology in a broad sense, that is, independent of the form of technology (eg, digital skills, experience with technology, computer literacy, and attitudes toward health technology), or related to a specific form of technology (eg, experience with VR).

### Adaptation

The adaptation strategies mentioned by the participants were grouped along the elements of the eHealth technologies that can be adapted to the eHealth user (Table 4), namely channeling (46/100, 46%), content (24/100, 24%), graphical parts of the technology (16/100, 16%), the functionalities (10/100, 10%), and the behavior change strategy that is used by the eHealth technology (4/100, 4%).

**Table 4:** Elements that can be adapted (N=100).

Customization	Themes, n (%)	Participants, n (%)
Channeling	46 (46)	8 (80)
Content	24 (24)	8 (80)
Graphical	16 (16)	8 (80)
Functionalities	10 (10)	4 (40)
Behavior change strategy	4 (4)	2 (20)

Strategies for channeling the eHealth message were related to adapting the way the eHealth technology was delivered. Participants mentioned channeling as a strategy (46/100, 46%), with several ways of adaptation mentioned, namely including personal details in the messages, adapting the timing of the messages, adapting the way the messages were delivered, and adapting the technology used to deliver the eHealth intervention. To begin with, including personal details in messages was mentioned as a way to adapt an eHealth technology, ranging from including the first name, second name, or gender in the communication to the eHealth user. One participant associated this way of adapting the eHealth technology with the cocktail party effect:

There are some indications that personalized material increases someone’s attention to it so, the cocktail party effect, when we hear our name, we process that information differently.

Participant 5

Second, the timing of the delivery of eHealth technology was mentioned as a way of tailoring the channel to the eHealth user. Examples range from adapting the frequency of sending messages to the user, whether to send a reminder, when to send a reminder, and sending messages after an alarm has been generated for a certain value. An added value of timing the eHealth technology was mentioned by participant 8:

And well, if you’re able to shape that in a personal way, then I think it’s helpful and something that the human coach cannot do, especially when it comes to the timing. Yeah, at the moment, you feel tempted to eat unhealthily or to sit on the couch and do nothing or something, while actually it’s better to move at that moment, then the human coach is not there in general. But the technology can help you to, at that moment, make a healthier choice. [Participant 8] 

Third, adapting the way messages are delivered to the eHealth user is mentioned by participants as a way to adapt eHealth technology. Examples range from adapting the person delivering a message in a video, adapting the tone of voice to the eHealth user (eg, one that is more reflective and one that is more directive), the amount of information provided to the user, and the order in which different parts of the eHealth technology are provided:

...as professionals assume that a certain order is helpful or is logic or is this is the way we do it? This is how we’ve always done it. While a person using that application or that program may think otherwise, may think, well, I want to start with this. I want to start with relaxation. I don’t want to start with cognitive restructuring or whatever. So, I find it a bit of a struggle between what we think we know this is the way to do it in terms of order and allowing persons to do whatever they like at any point in time, which would be the ultimate personalization like this.

Participant 10

Finally, the medium used to deliver the eHealth technology is also mentioned by participants as an adaptation strategy for adapting the technology. Examples include using either an app or web-based eHealth technology, sending messages either by email or push messages via phone, or informing the user by text or video. An example is given by participant 5:

But maybe by offering it in different channels and then allowing people to choose whether they want to read, because that seems to be the only thing that they can do here, or maybe they want to use the information in another way or via video or audio. [Participant 5] 

Content was also mentioned by participants (24/100, 24%) as a part that can be adapted to the eHealth user. Adaptation of content ranged from receiving different content (eg, offering different therapeutic approaches), providing feedback related to user input (eg, comparing provided data with the eHealth user’s goal), comparing the eHealth user’s data with data from peers, and giving advice to the eHealth user based on the data provided by the eHealth user.

Graphical aspects of the eHealth technology were mentioned by participants as a part that could be adapted to the user (16/100, 16%). The examples given by the participants ranged from very simple adaptations (eg, changing the colors) to changing the layout of the technology to more complex adaptations, such as changing an environment in VR or creating avatars adapted to the eHealth user:

And people can also develop their own avatar, which, of course, gives already a nice personalization aspect to it.

Participant 9

Functionality was also mentioned by participants as a part that can be adapted to the eHealth user (10/100, 10%). This is related to including or excluding functionalities (eg, in or excluding functionality that allows inviting others as social contact) of the eHealth technology based on the data provided by the eHealth user. Finally, 4% (4/100) of the participants mentioned examples of adapting the behavior change strategy to the user. This refers to adapting the way in which the eHealth technology aims to change behavior, for example, by providing different persuasion strategies according to the characteristics of the eHealth user. An example was given by participant 2 where, depending on the data provided by the user, gamification is only used as a way to change behavior if it is an appropriate strategy for that person:

And I think sometimes, and there’s been lots of work on, for example, whether or not gamification has added value. And the general feeling is that, yes, it does have some added value. But it’s pretty small still. And I think that, at least, partially, because it works well for some and not so for others.

Participant 2

### Matching

Participants mentioned substantiation methods (24/56, 43%) and variable level (32/56, 57%) as 2 components for matching the segmentations with adaptations (Table 5).

**Table 5:** Parts that can be adapted (N=56).

Matching	Themes, n (%)	Participants, n (%)
Substantiation	24 (43)	6 (60)
Variable level	32 (57)	7 (70)

Participants identified several methods of substantiation for matching segments of eHealth users to adaptations of eHealth technology. These are pilot studies (12/56, 21%), using theory for matching (6/56, 11%), conducting interviews (4/56, 7%) and the use of AI to derive insights specifically for individual users (3/56, 5%). First, participants mentioned several forms of pilot studies as examples of how segmentation and customization can be matched. These are studies in which a (prototype) version of the eHealth technology is available for use and is used as material during the study. Examples of pilot studies mentioned are matched study design and asking users to rate which messages they like within the eHealth technology:

So, I did a large surveys with, you know, 500 people, ask them to rate certain messages and how motivated they thought these messages were or not, could also be demotivating. And then also asked their personality and of, course, age and gender, and other demographic information. Based on that I found, you know that certain messages fit better with certain personality types, and certain genders.

Participant 7

Mixed methods pilot studies were also mentioned by participants. For example, one way to match segmentation and adaptation is to pilot an eHealth technology and interview users who are congruent with the intended use and people who are not congruent with the intended use. In this way, users’ views on why a technology (does not) fit their characteristics can be translated into a personalization or adaptation strategy. Another example of a pilot study was given by participant 2:

So, what you can do is get people to try out different versions of an intervention and just measure their response and engagement and as engagement, is a predictor of effectiveness, we could also use, well, the version of the intervention that provokes the most engagement to an individual might also be the version that’s most, that’s best personalized to them. And that has the highest chances of being effective for this individual.

Participant 2

Using an existing theory or developing a theory for a personalization or tailoring strategy was also mentioned by participants (5/56, 9%) as a way to match the segmentation and adaptation strategy of an eHealth technology. An example of using existing theory is to include segmentation variables that have been found in previous research to be predictors of target behavior.

...you need to have a very strong mixed methods approach, you really need to, so maybe if I write a few things down here. But any intervention on health should be based on a logic model, a logic model, since you really need to have a clear theory of what you're doing, and you need really to understand the issue...

Participant 3

The use of interviews means that data are collected in an open format and then translated into a segmentation and adaptation strategy (4/56, 7%). Participants mentioned that one way to gain information about a strategy is to explore whether there are differences within the target group during the interviews or to ask explicitly about their preferences. Participants mentioned that because of the open nature of the interviews, it is possible to go to go in more depth while gaining information about the strategy than with other methods; therefore, the personalization or tailoring strategy can also be explored in more depth. In addition, another added value mentioned was that segmentation variables or tailoring strategies can be developed, which was an aspect the developer or designer of the eHealth technology had not previously thought of. In an example related to interviews, the participant described that part of the user group expresses different needs in the way they could navigate through the eHealth technology:

And from the interviews with them, we found out that it was more that they were looking for someone who took them by the hand and did everything step by step. So how we translated that in the intervention was that the only control they had in terms of navigating an intervention was clicking next and previous...

Participant 5

The use of AI to derive insights specifically for individual users was mentioned as a way to substantiate the matching of segmentation and adaptation (3/56, 5%). This way of creating an adaptation strategy means that at an individual level, it is decided what works for whom (adaptation strategy) using data science techniques. This is different from other substantiation strategies because other substantiation is more focused on what works for whom at a group level:

Well, for example, if I take an extreme example now, but just to make a case. So, the kind of research that I do is I can model individual behavior. So, we model your sleeping behavior for three weeks, and based on modeling your own behavior, we design interventions that fit your pattern...

Participant 3

The use of guidelines was least mentioned by participants (2/56, 4%) for matching segmentation and adaptation. One example mentioned by participant 6 was related to the customization of the treatment, which needs to be in line with health care criteria and guidelines. In addition, the same participant mentioned working with dietitians to find guidelines for adapted dietary recommendations for the user.

In addition to “substantiation,” the second main code related to the theme “matching” was “variable level.” Participants mentioned several variable levels to match the segmentation with the adaptation strategy, namely grouping variables (18/56, 32%), direct input (8/56, 14%), and per variable (6/56, 11%).

Participants mentioned several ways in which variables can be grouped to personalize eHealth and varied in how these methods can be applied (18/56, 32%). First, they mentioned that users can be grouped into smaller segments using multiple segmentation variables. Personas and profiles are 2 specific examples that were mentioned. Personas consist of groups of users with similar characteristics across a diverse range of variables (eg, similarities in demographics, eHealth literacy, and preferences), whereas profiles consist of segments that are described on multiple variables associated with a particular concept, such as lifestyle profiles or risk profiles.

Participants gave 2 examples of how these personas and profiles can be used. First, personas can be created at the beginning of the eHealth development process, and these different personas can then be translated to develop an adaptation strategy that matches the characteristics of the persona descriptions. Second, these profiles or personas can be used within the eHealth system to segment the eHealth users to whom an adaptation strategy is applied. Participant 5 described this use of personas and profiles:

We assume it’s personalized because it’s personalized to the persona. But the persona is a fictional representation of a group of individuals. So, it’s not personalized to the individual. It’s personalized to individuals like that person, as seen by the designer or researcher.

Participant 5

In addition to using representatives for groups of users, 1 participant mentioned that variables can also be combined to create profiles similar to “Facebook profiles,” where each user is unique. This can be translated into a continuum where personas and profiles can be created from a variety of variables on the individual level to a group level. Participant 5 described how one can decide at what level these personas and profiles can be created to allow for tailoring or personalization:

To the degree that they show maximum diversity between the groups and maximum homogeneity within the groups. And I would do that and have done that in a data driven way.

Participant 5

Direct input from the user was also mentioned by the participants as a way of matching the segments with adaptation strategies (8/56, 14%). One participant mentioned that this way of matching segmentation and adaptation is not included in the model by Hawkins et al [15], which assumes that the developer of the eHealth technology collects information about the user and translates it into an adaptation strategy. The use of direct input means that the user adapts the eHealth technology themselves, and no information about the user is collected beforehand for segmentation. Participant 5 described how this can be applied:

So that cuts out the middleman, so you no longer have to measure something because you’re both the person that is being measured and the one that is adapting. So, if someone is going to choose the color of their phone, they don’t have to ask themselves what is your favorite color and then process or produce that fitting phone case, they can immediately make one themself or choose one themselves...

Participant 5

Finally, participants mentioned that segments and adaptation strategies can be matched per variable (6/56, 11%). This means that users are segmented on 1 variable and that adaptation strategies are developed on that single variable. Participants mentioned that this can either be done on an absolute measure of variables (eg, a segment on the variable name and matching this one variable with the inclusion of a name in messages) and that changes to this single variable can be used to match segmentation and adaptation:

So, I think you have to also adapt immediately to changes in the data. I think that’s one way to personalize and get well; then you don’t even need groups, I think, to form groups.

Participant 6

Moreover, participant 5 mentioned that matching per variable is mainly useful for adaptation strategies related to the content of the eHealth intervention:

It’s a different extent in whether it’s on the level of the individual. The thing is, I know that you are able to generate a tailored message using computer tailoring, for example, that you measure psychological constructs, and on the basis of that, you use information and look at the message database to construct a completely unique message for the individual. And I like this principle. I think it works for content, but I think it doesn’t work for graphics, channel, and source.

Participant 5

## Discussion

### Principal Findings

In this multimethod qualitative study, we sought to gain insights into the definitions and distinguishing factors of personalization and tailoring, their two components (segmentation and adaptation), and how these two are matched.

We identified 16 unique definitions of personalization and 28 unique definitions of tailoring in the literature and through interviews with eHealth experts. Some definitions focused on the more general description of “adaptations,” “individual users” or “user groups,” while other definitions described personalization and tailoring in terms of how segmentation and customization can be operationalized. Similar outcomes, such as increased motivation, relevance and persuasiveness, were described in definitions of tailoring and personalization, reflecting an optimistic perspective. Interestingly, none of the definitions describe outcomes that are related to the adverse effects of applying these concepts.

We found that definitions of tailoring included the term “computerization” or related terms, such as “decision rules,” whereas definitions of personalization did not. Although the definitions do not include a description of how these decision rules should be established, the inclusion of terms such as “prepared texts” [48] implies that decision rules for tailoring are developed in advance and that these decision rules are not established based on the data collected from the user. We also observed that definitions of personalization from the literature regularly included “identification,” while definitions from eHealth experts did not include this in their definitions. Moreover, we observed that “identification” is less common in definitions of tailoring and that the definitions of tailoring that do include “identification” describe it as an umbrella term for personalization and other strategies (such as feedback).

Along with the definitions of tailoring and personalization, we sought to gain insights into how they can be applied to eHealth technologies using an existing framework [15] expanded with “matching” to describe how segmentations and adaptations are linked in a meaningful way. We found that eHealth users can be segmented based on their demographics (eg, age), preferences (eg, like or dislike an exercise), health (eg, symptoms), psychological variables (eg, distress), behavioral variables (eg, physical activity), determinants (eg, self-efficacy), environmental information (eg, whether the user is at work), intervention interaction (eg, number of visits), and technology (eg, eHealth literacy). These segmentation variables can be matched with adaptations, where the adaptation reflects 1 variable, a group of variables, or the direct user input.

We identified 5 elements of eHealth technologies to which adaptations relate, namely channeling (eg, including one’s name in feedback messages), content (eg, providing normative feedback), graphical (eg, avatars that look similar to the user), functionalities (eg, including or excluding self-monitoring), and behavior change strategy (eg, including or excluding certain persuasion strategies). The match between the segments and customizations can be based on pilot studies, theory, interviews, AI for deriving individual-level insights, and guidelines. During the interviews, participants mentioned that these substantiations contribute differently to the matching of segmentation and customization. On the basis of the interviews, we have summarized the substantiation methods for matching segmentation and customization with a description of their objectives and examples of research questions in Table 6.

**Figure 1:**
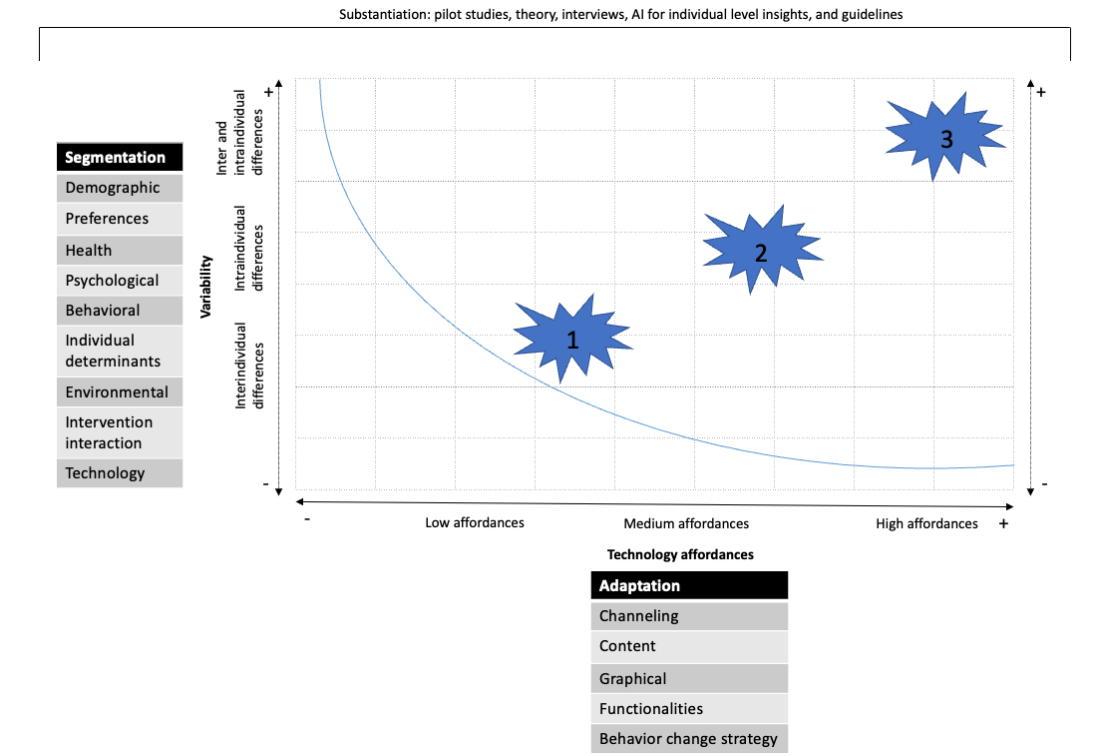
Model summarizing how variability and affordances are related.

Overall, we observed that variability and technology affordances seem to determine whether and how personalization and tailoring should be applied to eHealth technologies, according to participants. In Figure 1, we illustrate how these variables may be related to each other according to the participants. Variability in segmentation variables (shown on the y-axis) may be interindividual (such as differences in country of birth), intraindividual (such as changes in emotions), or both intra and interindividual (eg, a combination of emotions and country of birth).

**Table 6:** Substantiation methods for matching segmentations and adaptations.

Method	Goal	Examples of research questions
Pilot studies	Test an existing (prototype version of) segmentation and customization strategy	Which version of the eHealth technology fits best with which user segment?
Theory	Form a link between segmentation and customization	The target group is very different in terms of technology skills, how can we adapt our technology?
Interviews	Form hypotheses about which customizations must be included in the eHealth technology	Does the target group express different needs or preferences during the interviews?
AI for deriving individual-level insights	Develop customizations on the individual level	What patterns of behavior do we observe at an individual level, and how can we adapt the eHealth technology to these individual patterns?
Guidelines	Gather information on segmentation and adaptation at the group level	What kind of treatment should the eHealth technology offer to users with high blood pressure?

In addition, the affordances of the technology (x-axis) determine which adaptation strategy can be used. For example, a technology with high affordances such as VR allows adaptations in terms of channeling, textual content, graphical aspects, functionalities, and behavior change strategies, whereas an SMS text messaging–based eHealth technology can only be adapted in terms of textual content, channeling, and behavior change strategy. The area under the blue line depicts the area in which there is a mismatch between segmentation variability and technology affordances, resulting in ineffectively applied personalization and tailoring in eHealth technologies. The stars represent the desired combination of segmentation and adaptation levels, which, according to the participants, should ideally be at the same level. This means that low levels of segmentation should be combined with low levels of adaptations and the other way around. The illustration of each star is mentioned subsequently.

The first star represents the target group of the eHealth technology that shows interindividual differences (differences between users), and the technology with medium affordances allows for adaptations on the same level. An example is an eHealth technology that collects the name of the user (interindividual differences) at the first visit. On subsequent visits to the eHealth technology, a text is displayed saying “Welcome, John!” Another example is to segment users based on their visual appearance (interindividual differences) and to adapt characters in a virtual environment by making them look similar. As these examples illustrate, data can be collected once (or on a very long interval) because intraindividual differences are small, for example, through questionnaires and qualitative data collection.

The second star represents the target group of the eHealth technology that has intraindividual differences (differences within users), and the technology needs medium affordances to adapt to these differences over time. An example is an eHealth technology that segments users based on their location. If the eHealth user is in an environment where there is a place nearby for possible physical activity, the eHealth technology sends a message (channeling) to remind him or her to go for a run. As shown in this example, data must be collected multiple times to allow adaptations to these intraindividual differences, for example, through ecological momentary assessment, sensor data, log data, and activity trackers. The eHealth technology must have the affordances to translate these data into adaptations over time.

The third star represents the context in which eHealth users show both inter and intraindividual differences. An example is an eHealth technology that segments users by both their communication channel preference and the number of steps they take per day. Users indicate whether they want to use an eHealth technology on their computer or their mobile phone, through which it is then used. In addition, users receive motivational messages based on the number of steps, if this number is <2000. If the number of steps is >2000, the user will receive reinforcement messages. As shown in this example, data must be collected once for interindividual differences and several times related to intraindividual differences. The eHealth technology needs to have high affordances to allow adaptations that are stable and adaptations that occur over time.

To illustrate, one may use the theory about eHealth literacy to tailor or personalize the eHealth technology. However, if there are no intra or interindividual differences in eHealth literacy (low on the y-axis), there is no added value in segmenting and adapting based on this concept. Moreover, if an eHealth technology has very low affordances, such as an SMS text messaging–based eHealth technology, there is a low allowance for applying the different adaptation strategies (low on the x-axis); therefore, segmentation on multiple inter and intraindividual variables will often be obsolete.

### Theoretical Implications

We argue that we can arrive at a clear definition of tailoring and personalization by merging the definitions from health communication research (mainly used in eHealth evaluation studies) with more recent definitions from other fields and eHealth experts. We observed a difference in the adaptations that are included in the definitions: while personalization shows a stronger focus on identification (including individual values) tailoring shows a focus on channeling and computerization (decision rules). We argue that the pattern underlying this difference cannot be captured in terms of segmentation, adaptation, and outcomes but in the way they are matched. While tailoring involves decision rules to match information collected about the individual user with general characteristics of groups, personalization does not include this in its definitions, as previous work in health communication research has focused on inserting identifiable aspects of the user. The affordances of the channels used in health communication research (brochures, leaflets, and later websites that largely mimic paper health information) were limited to adapting textual aspects and did not collect data from the user in real time, so definitions of personalization are limited to inserting someone’s name or raising expectations. Nonetheless, advances in technology are broadening the area in which personalization can occur (more than just including one’s name or raising expectations). The ability of technology to collect large amounts of data in real time and the potential of algorithms to extract meaningful insights from those data allow for individual-level matching at a deeper level than only “inserting” data from the user in communications. Therefore, based on published definitions and interviews with eHealth experts, we have formulated the definitions of eHealth personalization and eHealth tailoring as follows*:*

Segmentation of eHealth users based on (a combination of) demographics, preferences, health, psychological, behavioral, determinants, environment, intervention interaction, technology, matched with adaptations to content, channeling, graphics, functionalities, and/or behavior change strategies... by inserting this individual (segmentation) information or by translating this data into individual-level insights that are in turn translated into adaptations.

eHealth personalization

...by coupling segmentation variables with general characteristics of people with similar data using if-then algorithms.

eHealth tailoring

Both personalization and tailoring can be user-initiated, where this is referred to as customization in the case of personalization (technology has affordances for the user to directly change technology itself) and as self-tailoring in the case of tailored eHealth, where the user specifies their preferences or other information from which preferences are inferred using if-then algorithms.

### Practical Implications

The literature shows mixed results on the effectiveness of tailoring and personalization. For example, the inclusion of a personalized meal planner [56] or tailoring for smokers with a low socioeconomic status [57] did not show better outcomes compared to a similar eHealth technology. In contrast, tailored advice on action planning [58] and daily tailored feedback on energy and fat intake [59] led to better outcomes. At this stage, it is difficult to draw conclusions about the effectiveness of personalization and tailoring because all the forms (all different segmentations, adaptations, and matching) are put together. It may be that one form works better than the other, which could explain the mixed results in effectiveness. We therefore suggest that the use of personalization and tailoring be reported in terms of segmentation, adaptation, and matching. By focusing reporting on segmentation and adaptation strategies (per “matched” segmentation and adaptation) with a description of how these were matched, we can compare and combine different forms, and subsequently, we may gain more insights into the working mechanisms of tailoring and personalization. For example, we can compare which theory gives the best results for building a segmentation and adaptation strategy, which segmentation variables are most relevant for personalizing and tailoring eHealth technologies, and which adaptation strategy increases the effectiveness of eHealth technologies. The items in Textbox 1 could be included as an extension of the CONSORT (Consolidated Standards of Reporting Trials)-EHEALTH checklist [60] as a first step to gaining a better understanding of the working mechanisms of personalization and tailoring. This checklist is broader than the reporting standards described by Harrington and Noar [61], as it can also be used to report on channel adaptations, graphical properties, functionalities, and behavior change strategies.

**Suggested reporting standards for tailoring and personalization approaches in eHealth.**

**Segmentation and customization (description per “matched” link of segmentation and customization)**

- Name the variables used to segment users into more homogeneous groups and which data collection method was used to collect data for segmenting eHealth users through the eHealth technology.
- Name which part of the eHealth technology was adapted (channeling, content, graphical, functionalities, and behavior change strategy) and how this part was adapted to represent the user segments.
- Describe how the match between segmentation and adaptation was substantiated (give details on the theory, pilot studies, artificial intelligence for deriving individual-level insights, guidelines, or interviews).
- Clarify the variable level through which segmentation and adaptation are linked (direct input, per variable, and grouping variables) and provide, where applicable, details about the algorithm or grouping that was used.

### Limitations

An important limitation of this study is the lack of generalizability. Besides reviewing the literature for definitions, we have interviewed 10 eHealth experts through convenience sampling. As the context in which eHealth technologies use segmentation and adaptation is of great importance, it is not clear whether we have gained insights into these contextual factors and consequently, the different ways in which segmentation and adaptation are applied to eHealth technologies. However, we did include vignettes that are related to different contexts so that participants were able to provide input on eHealth technologies related to other contexts.

### Conclusions

Overall, we found that personalization and tailoring are multidimensional concepts with multiple factors at play that determine how these concepts should be applied to eHealth and how effective they are. For example, continuously providing personalized feedback on segmentation variables that remain relatively stable over time may become more irritating than helpful, reducing the sense that the eHealth technology is truly personalized or tailored to the user. In short, some behaviors may require complex and idiosyncratic adaptations that personalization can provide. This requires a different study approach; while randomized controlled trials are appropriate for the development of if-then algorithms for tailoring, the development of personalization requires other study designs, such as an N-of-1 trial.

## Appendix

Multimedia Appendix 1 Interview scheme.

Multimedia Appendix 2 COREQ (Consolidated Criteria for Reporting Qualitative Research) checklist.

Multimedia Appendix 3 Tailoring definitions.

Multimedia Appendix 4 Personalization definitions.

## Abbreviations

CONSORT: Consolidated Standards of Reporting Trials
COREQ: Consolidated Criteria for Reporting Qualitative Research
VR: virtual reality

## Data Availability

The datasets generated during and analyzed during this study are not publicly available due to the absence of explicit consent from participants but are available from the corresponding author on reasonable request.
