# Supplementary material for: Clarifying the Concepts of Personalization and Tailoring of eHealth Technologies: Multimethod Qualitative Study
Source: J Med Internet Res. 2024 Nov 13;26:e50497. doi: 10.2196/50497 (PMC11602766; doi:10.2196/50497)
Supplement: Multimedia Appendix 1 [file jmir_v26i1e50497_app1.docx]

## Multimedia Appendix 1: Interview Scheme

Introduction

First off, thank you for being here and letting me interview you. To give you a bit context, I’m trying to bring clarity into the definition of personalization in regard to eHealth technologies. Specifically, getting insight into how several forms of technology can be personalized and furthermore on which characteristics of an individual one should focus on to improve the personalization of eHealth technologies. To do that, I will first ask you a few questions regarding personalization of eHealth technologies. Then I will show you three vignettes, which in this case are a description of an eHealth technology, and I want you to give me your opinion for each of them, how you would personalize them and to which segments of the users. Any questions so far?

To start off with, please tell me a bit about yourself, your age, gender, your profession as well as any other experiences you have in regard to eHealth that you think might be relevant.

(1) Personalization

Since we are conducting a review to clarify the concept for personalization, how would you, from your perspective, define personalization in the field of eHealth?

Do you think there are differences between personalization and tailoring or any other similar terms, such as targeted, individualized, adapted?

Are you aware of any other similar terms that relate to personalization? How do you view them in relation to personalization?

What do you feel are the most important advantages of personalizing eHealth interventions? What are the most important disadvantages?

Have you made use of or applied personalization to eHealth technologies, and can you give an example?

How would you segment the users of eHealth technologies to provide them with personalized content?

To what degree would you segment the users? (how big should the groups be? You can use examples)

How would you collect the data from the user that you base the segmentation on? Automatic or manually?

How long would you continue to collect the data from the users and sort them into more specific segments based on that?

In what intervals would you collect the data?

How can eHealth technologies be customized to the end user? Does that vary over different forms of technologies and if yes how so?

Should inferences/ assumptions be made based on the collected data?

To what degree should the technology be customizable? Would it distract from its intended purpose?

How do you think customization and segmentation are related?

Can you think of any other variables that play a role in personalization, other than customization and segmentation?

(2) questions for during the vignettes:

What is your first impression regarding this technology? Which part would you personalize to make it more effective?

How would you segment the users of this technology? Broader or more individual?

Would you put it out there for people to find who need it or do you think it would be more effective if it would be delivered to certain groups of people? (like people that take a certain medication)

How much customization do you think would be effective for this intervention?

Should inferences in this case be made about the users’ data?
