# Supplementary material for: Clarifying the Concepts of Personalization and Tailoring of eHealth Technologies: Multimethod Qualitative Study
Source: J Med Internet Res. 2024 Nov 13;26:e50497. doi: 10.2196/50497 (PMC11602766; doi:10.2196/50497)
Supplement: Multimedia Appendix 3 [file jmir_v26i1e50497_app3.docx]

## Multimedia Appendix 3: Tailoring definitions

| **Reference (n)** | **Definition** |
| --- | --- |
| de Vries and Brug [1], 1999 | Computer-tailoring in health education can be defined as the adaptation of health education materials to one specific person through a largely computerised process. Computer-tailoring is more than personalised materials. |
| Kreuter and Wray [2], 2003 (11) | Tailored health communication can customize the source, message and channel of a given communication to a given individual, presumably maximizing the relevance of the communication to that person. |
| Kreuter et al [3], 2000 (8) | Any combination of strategies and information intended to reach one specific person, based on characteristics that are unique to that person, related to the outcome of interest, and derived from an individual assessment.” |
| Hawkins et al [4], 2008 (7) | Tailoring’ refers to any of a number of methods for creating communications individualized for their receivers, with the expectation that this individualization will lead to larger intended effects of these communications |
| Krebs et al [5], 2010 (4) | "computer-tailoring” is a method of assessing individuals and selecting communication content using data-driven decision rules that produce feedback automatically from a database of content elements.” |
| Lustria et al [6], 2009 (4) | Tailoring is a multi-dimensional communication strategy aimed at increasing the perceived personal relevance of health messages, which consequently, helps create ideal environments for persuasion (and behavior change) to occur. Computer-tailoring enhances the creation of tailored messages by facilitating the collection and assessment of individual data and then using evidence-based decision rules to create strategic health messages |
| Brug et al [7], 2003 (4) | Computer-tailored interventions mimic, to a certain extent, a classic tailoring technique, that of “person-to-person” counseling. As in counseling, computer-tailored interventions provide people with information that is based on their individual characteristics (eg, their behavior, attitudes, and perceived barriers), which makes the information personally relevant. In computer-tailored interventions, the diagnostic and educational expertise and techniques of the counselor are documented in a computerized expert system. |
| Oinas-Kukkonen and Harjumma [8], 2009 (3) | Information provided by the system will be more persuasive if it is tailored to the potential needs, interests, personality, usage context, or other factors relevant to a user group |
| Rimer and Kreuter [9], 2006 (3) | Tailoring could enhance motivation to process health information in at least four ways: (a) match content to an individual’s information needs and interests, (b) frame health information in a context that is meaningful to the person, (c) use design and production elements to capture the individual’s attention, and (d) provide information in the amount, type, and through channels of delivery preferred by the individual, thus potentially reducing barriers to exposure of individuals to communication interventions. |
| Kroeze et al [10], 2006 (2) | A computer-tailored intervention mimics interpersonal counseling using a computerized process, but, unlike interpersonal counseling, it can be widely distributed through interactive media channels at a relatively low cost. Computer tailoring allows for individualized feedback and advice on personal behavior, personal motivation, outcome expectations, self-efficacy, social and physical environmental opportunities, and other behavioral determinants. |
| Brug et al [11], 1999 (1) | The process of computer tailoring is similar to person-to-person patient counseling. Individual subjects are interviewed or surveyed (‘screened’) and the results are used to develop an individual treatment plan, behavior change plan or dietary advice. In computer tailoring the expertise of the nutrition educator is programmed into a computer which reads the (coded) survey responses and generates individualized feedback messages. |
| Smeets et al [12], 2007 (1) | Computer tailoring is a technique in which information on health behavior change is tailored to the individual |
| Bental et al [13], 1999 (1) | We consider tailoring that adapts the content of the material or the way that the content is presented according to the needs of the individual |
| Nahum-Shani et al [14], 2016 (1) | Various scientific fields have used different terms to describe interventions that adapt the provision of support to an individual’s changing internal and contextual state. These include dynamic tailoring [17], intelligent real-time therapy [18], and dynamically and individually tailored ecological momentary interventions [19]. Here, we use the term JITAI because it integrates two concepts: “just-in-time” and “adaptive” |
| Păsărelu et al [15], 2017 (1) | Individually tailored treatments are those interventions that combine modules from different treatment packages, targeting multiple disorders […], in the tailored condition, participants receive a different treatment based on the patients’ unique symptom presentation, preferences, and characteristics (e.g. education level, motivation) |
| Strecher [16], 1999 (1) | To tailor materials, one must first collect information from an individual in order to create a program designed to meet his or her specific needs. Referred to in a variety of ways (e.g., ‘‘personalized materials’’, ‘‘expert systems’’, ‘‘mail-merge on steroids’’), tailored materials require: (a) collection of characteristics, at an individual level, relevant to smoking cessation (or movement through stages of change), (b) an algorithm that uses these data to generate messages tailored to the specific needs of the user, and (c) a feedback protocol that combines these messages in a clear, vivid manner |
| Smit et al [17], 2015 (1) | Based on this definition, we define message frame tailoring as adjusting this perspective (highlighting some bits of information while omitting others) based on people’s individual needs. |
| Nguyen et al [18], 2016 (1) | In this study, we refer to “mode tailoring” as adjusting information to match individual preferences for presentation modality, using verbal (text), visual (static illustrations), and/or audiovisual (videos) information |
| Dijkstra [19], 2008 (1) | Computers make it possible to compose a persuasive text for the individual. To do so, the computer needs specific information about the individual (Figure 1). Furthermore, the computer program includes decision rules that use the information on the individual to compose the persuasive message. The composition may comprise a selection of pieces of prepared texts that are available in the text library, and it may directly include information about the individual (e.g., the person’s name). The resulting computer-tailored information may be offered to the individual through different channels (e.g., on screen, printed) |
| Heron and Smyth [20], 2010 (1) | EMI can be tailored to participants in two ways. First, the content of the EMI can be specifically designed based on information individuals provide during pre-intervention assessments or momentary assessments (i.e., EMA). About two-thirds of the studies (k=19) used EMI with tailored content, three (11%) include a combination of tailored and generic messages, and five (19%) used only generic messages (Table 2, EMI Content Tailored). The tailored EMI included interactive CBT activities (e.g., relaxation, problem solving, goal setting), feedback based on concurrent EMA, individual counseling, or messages based on pre-intervention behavior patterns. The generic content EMI were generally motivational statements or reminders to practice learned skills (e.g., CBT).  A second method of tailoring EMI involves delivering the interventions at specific moments when individuals are especially in need of additional support. In order to provide time tailored EMI, the events or circumstances that trigger or initiate the delivery of the intervention must be clearly defined. A variety of delivery protocols were utilized that included user initiated, fixed prompt times, random prompted times, and tailored dates and/or times (see Table 2, EMI Triggers). EMI that were user initiated relied on participants to initiate contact with the electronic device when specific events occurred (e.g., ate a meal, exercised, experienced anxiety, craved a cigarette). Fixed and random prompt times involved the palmtop computer or mobile phone providing an auditory alarm signaling the receipt of an EMI at either specific pre-arranged times when participants were notified they would receive messages, or at seemingly random times (i.e., participants did not know when they would receive the EMI).” |
| Participant 1 | Yeah, and I think there you have the same thing because you can say, um, targeting and tailoring that I see that also in these dimensions and you can say targeting is for example, if I do it more specifically focused on women or men or lower educated or higher educated or what have you. So that's I think what's classically is called more targeting and with tailoring you know more. But like when is it like if I know that you are a female and you're highly educated and you have this background, ethnic background and you have this preference, like when do you call it then tailoring? Because I think it's just the more you know, the more fine grained you can do it. And in the end, the more you know, the more options you have. So then you can make it more so if you can say, OK, I want to know your gender, your age or ethnicity, your job status and so on, but also more psychological variables like attitude and self efficacy and so on. These are all options, right? So even if you had only to like, say, male, female, and so you have two times two times two times two. So in the end, you end up with so many possible combinations that it becomes almost that every person gets a different intervention. But it's not because there are so many different interventions, but there are so many possible options. |
| Participant 2 | Yeah, well, to me, it's more of a gliding scale, what I said before, so I think tailoring is more often used to still refer to groups to tailor to a certain group of people. Whereas I would say personalization is more towards an individual. But there's also of course, a gliding scale, because the smaller you make your groups the closer you get to personalization, in my view. And the second thing is, it's probably not inherent in the word tailoring. But the way it's used, it's often within health psychology, tailoring is very often focused only on the content of an intervention. So what kind of messages you give what kind of information is relevant to a certain target group? Whereas I think personalization can be seen as a little bit more encompassing. So also the technology itself and the way you deliver it and things like that. And I think that that could also be included in the concept tailoring but very often isn't at this moment. |
| Participant 3 | but for me, tailoring is involving a allocation decision that is made by an external agency or by an algorithm. Whereas personalization also involves personalisation by choice. I find tailoring terms of a narrower, which really depends on that other people are making decisions about you or other processes make decisions about you. Well, I think in personalization, I would involve things things like being able to click I never want to see that again. |
| Participant 5 | So if someone said to me, hey, [name], check out all your deals. So that is targeting or segmentation and tailoring is assumed to be more involved, more personalized to the individual. So maybe that more things are adapted so more aspects of the individual are measured and there is adaption going on not just in name, but also in the psychological constructs targeted, in the functionality and the look and feel of the intervention. |
| Participant 6 | But I think in English, yeah it will, maybe it differs a bit that personalized is actually a more, even more fitting to the person then, for example, tailored. That tailor is to make adjustments or something and that personalized is really, that thing that really fits the person, the individual. So really moving away from the, for example also in research settings, moving away from the large randomized control trials where you are looking for a mean, or a general. Yeah, the mean, mean person doesn't exist. So yeah, what, then you should actually study those persons. individually. I think so n of one studies I'm really interested in. And I think if you mentioned tailored medicine, then you're still looking for those outcomes of randomized control trials and try to adjust. And personalized i think is it starts from another point of view that you really are starting from the individual. Yeah. Hope that's a bit clear. |
| Participant 7 | And so I think the distinction that most people make between personalization and tailoring is that tailoring is more towards groups or characteristics of groups. And personalization is more towards individuals. I can follow that definition as well, I think that's fine. If we agree upon the definition that would be fine with me. Targeting, Im not super familiar with what people usually use as the definition. But I guess it's similar to tailoring when you target a group or characteristic of a group. |
| Participant 8 | Yeah, when it comes to tailoring for us, for instance, then, well, what my image of tailoring is, is that it's more based on group membership, for instance. So the sender of the information knows at a certain point that you are a female student in your 20s at a technical university, and based on that information you are already capable of tailored information. So that's my image of tailoring. And I don't know whether that's correct. But when it comes to personalization, it's a higher level of tailoring, so to say, because it's not that important anymore to which groups you belong. It can be used to make this personalization, to reach it sooner. So, to say, but at the end it's about you as a person and of course it's important maybe that you are a female student in your twenties, but that comes out of the data that are being gathered. And that that is the basis, I would say, of personalization. For tailoring I would say it's not that and well, to be honest, also technology and data science and artificial intelligence is not yet able to reach personalization at the highest level, I think, or it takes a lot of time, this training period before. So, in the beginning still it's important to make some sort of profile or something. |
| Participant 10 | Well. What maybe I don't know, I think this is partly semantics, but what my first association thinking about these terms is that when we talk about targeting, this hints at a certain ambition of reaching a certain group of people and thereby making sure that you use the right channel and you use the right messages, et cetera. While I think personalization is more a consumer perspective, is like, how can I make this technology my own. How can I make it work for me? So, I think the words come from different, maybe different angles to some extent. At least in my view, I would consider personalization typically something that is very much consumer oriented. It's like, what do people need? What do they like? While targeting and tailoring seems to me like the you know, the professional thinking about how can I make them, it has a more sort of manipulative sound to it. Also, a connotation of I want to do something to them, how do I reach them and what do they like? It's maybe more the Coca-Cola and other companies thinking about how can I tailor it, how can I target them, how do I get them to do what I would like them to do. But this is just my personal sense of the difference, maybe in terminology. |

1. de Vries H, Brug J. Computer-tailored interventions motivating people to adopt health promoting behaviours: Introduction to a new approach. Patient Education and Counseling. 1999;36:99-105.

2. Kreuter MW, Wray RJ. Tailored and Targeted Health Communication: Strategies for Enhancing Information Relevance. Am J Health Behav. 2003;27:S227-S32.

3. Kreuter M, Farrell D, Brennan L, Olevitch L. Tailoring Health Messages: Customizing Communication With Computer Technology. New York, NY: Routledge; 2000.

4. Hawkins RP, Kreuter M, Resnicow K, Fishbein M, Dijkstra A. Understanding tailoring in communicating about health. Health Educ Res. 2008 Jun;23(3):454-66. PMID: 18349033. doi: 10.1093/her/cyn004.

5. Krebs P, Prochaska JO, Rossi JS. A meta-analysis of computer-tailored interventions for health behavior change. Prev Med. 2010 Sep-Oct;51(3-4):214-21. PMID: 20558196. doi: 10.1016/j.ypmed.2010.06.004.

6. Lustria ML, Cortese J, Noar SM, Glueckauf RL. Computer-tailored health interventions delivered over the Web: review and analysis of key components. Patient Educ Couns. 2009 Feb;74(2):156-73. PMID: 18947966. doi: 10.1016/j.pec.2008.08.023.

7. Brug J, Oenema A, Campbell M. Past, present, and future of computer-tailored nutrition education. Am J Clin Nutr. 2003 Apr;77(4 Suppl):1028S-34S. PMID: 12663313. doi: 10.1093/ajcn/77.4.1028S.

8. Oinas-Kukkonen H, Harjumaa M. Persuasive Systems Design: Key Issues, Process Model, and System Features. Communications of the Association for Information Systems. 2009;24. doi: 10.17705/1cais.02428.

9. Rimer BK, Kreuter MW. Advancing Tailored Health Communication: A Persuasion and Message Effects Perspective. Journal of Communication. 2006;56(suppl_1):S184-S201. doi: 10.1111/j.1460-2466.2006.00289.x.

10. Kroeze W, Werkman A, Brug J. A systematic review of randomized trials on the effectiveness of computer-tailored education on physical activity and dietary behaviors. Ann Behav Med. 2006 Jun;31:205-23.

11. Brug J, Steenhuis I, van Assema P, Glanz K, De Vries H. Computer-tailored nutrition education: differences between two interventions. Health Educ Res. 1999 Apr;14:249-56.

12. Smeets T, Kremers S, de Vries H, Brug J. Effects of tailored feedback on multiple health behaviors. Ann Behav Med. 2007 Jun;33(2):117-23.

13. Bental D, Cawsey A, Jones R. Patient information systems that tailor to the individual. . Patient Educ Couns. 1999 Feb;36(2):171-80.

14. Nahum-Shani I, Smith S, Spring B, Collins L, Witkiewitz K, Tewari A, et al. Just-in-Time Adaptive Interventions (JITAIs) in mobile health: key components and design principles for ongoing health behavior support. Ann Behav Med. 2016 Sept 23;52(6):446-62.

15. Păsărelu C, Andersson G, Bergman Nordgren L, Dobrean A. Internet-delivered transdiagnostic and tailored cognitive behavioral therapy for anxiety and depression: a systematic review and meta-analysis of randomized controlled trials. Cogn Behav Ther. 2017 Jan;46(1):1-28.

16. Strecher V. Computer-tailored smoking cessation materials: a review and discussion. Patient Educ Couns. 1999 Feb;36(2):107-17.

17. Smit E, Linn A, van Weert J. Taking online computer-tailoring forward. . Eur Health Psychol. 2015;17(1):25-31.

18. Nguyen M, van Weert J, Bol N, Loos E, Tytgat K, van de Ven A, et al. Tailoring the mode of information presentation: effects on younger and older adults' attention and recall of online information. . Hum Commun Res. 2016 Oct 21;43(1):102-26.

19. Dijkstra A. The psychology of tailoring‐ingredients in computer‐tailored persuasion. . Soc Personal Psychol Compass. 2008 Feb 21;2(2):765-84.

20. Heron K, Smyth J. Ecological momentary interventions: incorporating mobile technology into psychosocial and health behaviour treatments. . Br J Health Psychol 2010 Feb;15:1-39.
