# Supplementary material for: Clarifying the Concepts of Personalization and Tailoring of eHealth Technologies: Multimethod Qualitative Study
Source: J Med Internet Res. 2024 Nov 13;26:e50497. doi: 10.2196/50497 (PMC11602766; doi:10.2196/50497)
Supplement: Multimedia Appendix 4 [file jmir_v26i1e50497_app4.docx]

## Multimedia Appendix 4: Personalization definitions

| **Reference (n)** | **Definition** |
| --- | --- |
| de Vries and Brug [1], 1999 (15) | Personalisation means that a person’s name is listed (once or several times) on the information that he or she receives. However, the information is not adapted to the individual’s diagnostic, behavioural or motivational characteristics. Tailoring refers to the fact that the content of the information is adapted to an individual’s characteristics |
| Hawkins et al [2], 2008 (8) | Although tailoring overall is about various forms of individualization, personalization attempts to increase attention or motivation to process messages by conveying, explicitly or implicitly, that the communication is designed specifically for ‘you’ |
| Dijkstra 2005 Dijkstra [3], 2005 (5) | Personalization refers to incorporating recognizable aspects of a person in a general text. The recognizable feature or set of features refers undeniably to the person, e.g. the person’s first name or the combination of objective behavioral features such as the number of cigarettes smoked, the number of years the person smoked and the brand smoked. |
| Oinas-Kukkonen [4], 2009 (3) | A system that offers personalized content or services has a greater capability for persuasion. |
| DiClemente et al [5], 2001 | Personalized Feedback represents the most individualized type of feedback and is based on personal information provided by the individual using some type of assessment procedure. Providing feedback to an individual on level of risk for various types of problems as a result of genetic or psychosocial assessment would represent personalized feedback. However, there can be many variations on a theme for this type of feedback. Personalization, for example, can be based on different types of data used as the source of the comparison. Feedback can provide normative comparison to a reference group or ipsative comparisons where the information is self-referent. Providing information as to how one’s level of drinking alcohol compares to the national norms for individuals of one’s age and gender would be an example of normative, personalized feedback. Offering at one point in time or over successive assessment points a comparison of an individual’s temptation to drink with his or her confidence to abstain across different types of situations would be an example of ipsative, personalized feedback. The other important distinction that is relevant for personalized feedback relates to the specific content of the feedback. Some feedback is risk or |
| Evers et al [6], 2014 | Thus, offering specific physical, psychological or social treatment modules instead of a standard treatment package is considered a relevant next step in attaining a personalized healthcare framework in the area of psychological and psychopharmacological interventions and psychotherapy. |
| Participant 1 | […] And I think what we try to do with personalization somehow is kind of go from mass media to more try almost mimicking what you do with a counselor. Right. To make it as personal as possible with the idea, of course, that we cannot have a counselor for everybody for every problem. So, like trying to come close to the efficacy of having only really a one-on-one chat, but at the same time making it available for a lot of people. So, the kind of this tradeoff is there. |
| Participant 2 | Well, in some way, adapting, in this case, a digital health intervention to an individual. And of course, you can go from many degrees and it's probably more of a gliding scale from things as tailoring towards personalization where personalization is more focusing on the individual, [whereas tailoring would be in my view a little bit more focusing on groups. And what you see in literature is that sometimes tailoring is very often seen as adapting the contents of an intervention to a specific group.] |
| Participant 3 | Well, personalization happens if the timing, content, or mode of delivery of intervention components is dependent on the person their behavior, their context or a timeline that has been derived from their previous exposure, or behaviors. |
| Participant 4 | And personalized means that it is tailored to the uses of that kind of technology, but also to the context, not only about the user, the end user, but also how the technology is personalized and tailored to a certain context or situation. |
| Participant 5 | […] personalization for me is that a product to a certain extent is specific to an individual or a group of individuals. So, personalization can be as small as changing the color of your phone screen. And then you have a personalized variation of the generic device of mobile phone. It can be directed towards an individual, as an example, it can also be that something is personalized towards a group of people, that can be specific health information for women. It can be specific information for patients that need a new hip instead of a knee. So, all of that is personalization for me. As long as there is some adaptation going on that changes the product from generic to specific to an individual or group of individuals. It's personalization to me. |
| Participant 6 | Well, I think personalization means that you adjust or adapt your, maybe also your assessment and the treatment of patients in a way that it's really fits that person. So not care as usual and put it over everyone. |
| Participant 7 | So, I would say that when I use personalization, I would mean that there is something that is specific to that person or the group that the person belongs to that you use to personalize your communications. So that's quite a broad definition. |
| Participant 8 | Um, yeah, well, it's quite a difficult question, of course, but, when it comes to my work and the projects we are working on, then personalization is, well, in our context, it's always about helping people, at an individual level to make healthy choices. So, personalization is several aspects, I would say that people experience that they're being coached or supported as a person. So, it's directed at them and not in general. So, it's a lot about the subjective experience, I would say. And it should lead to actionable insights that can be applied to their personal life. But if you ask me what is your definition? Well, it's always, I would say when it comes to supporting healthy choices, the feedback that is being offered should be experienced as personal so that people think or experience or perceive that they are being spoken to in a personal way. It accounts to them and not per se to their fellow peers or something. |
| Participant 9 | Well, quite simple in a way, eHealth for me is ICT for health care purposes, and so that’s quite broad and if you talk about personalized eHealth, it is how to shape and design, intelligent design, technology to support the different stakeholders in health care. So, for me, it's also supporting the patient, but also health care professionals or next of kin et cetera. So, it can be different "mantelzorgers" as we say here in the Netherlands. So, it's supporting the whole ecosystem around patients. And for me, what I use is a person-centered eHealth information and communication technology. |
| Participant 10 | […] No, I think in our group, we've talked about personalization a lot as a concept. Again, you know, taking a more sort of pragmatic approach to what it is. Typically thinking about how do you suit the needs and how do you make it as much preference based as possible. |

1. de Vries H, Brug J. Computer-tailored interventions motivating people to adopt health promoting behaviours: Introduction to a new approach. Patient Education and Counseling. 1999;36:99-105.

2. Hawkins RP, Kreuter M, Resnicow K, Fishbein M, Dijkstra A. Understanding tailoring in communicating about health. Health Educ Res. 2008 Jun;23(3):454-66. PMID: 18349033. doi: 10.1093/her/cyn004.

3. Dijkstra A. Working mechanisms of computer-tailored health education: evidence from smoking cessation. Health Educ Res. 2005 Oct;20(5):527-39. PMID: 15701665. doi: 10.1093/her/cyh014.

4. Oinas-Kukkonen H, Harjumaa M. Persuasive Systems Design: Key Issues, Process Model, and System Features. Communications of the Association for Information Systems. 2009;24. doi: 10.17705/1cais.02428.

5. DiClemente C, Marinilli A, Singh M, Bellino L. The role of feedback in the process of health behavior change. Am J Health Behav. 2001 May 01;25(3):217-27.

6. Evers A, Gieler U, Hasenbring M, van Middendorp H. Incorporating biopsychosocial characteristics into personalized healthcare: a clinical approach. . Psychother Psychosom. 2014;83(3):148-57.
